# Practical Chemistry and Pharmacy

**Published:** 1893-11

**Authors:** 

**Affiliations:** LaGrange, Ga.


### PRACTICAL CHEMISTRY AND PHARMACY.

EDITED BY HENRY R. SLACK, M. D., PH. M., LAGRANGE, GA.

### HOW TO PRESERVE AMMONIUM CARBONATE.

Every physician who is so unfortunately situated as to be compelled to keep a stock of drugs from which to fill his own prescriptions knows the trouble it is to dispense good carbonate of ammonium. When dispensed according to the "U. S. P." in white translucent masses," it is one of our best cardiac stimulants, but in the friable lumps, or white powder it is a bicarbonate and simply an antacid. Now to keep this important chemical from changing I find a coating of paraffine very efficient. As soon as you receive your ammonium carbonate scrape off the white powder that invariably covers it, break into lumps of convenient size and dip into melted paraffine, being careful to get the lumps well covered. Then as soon as cool put into a glass stoppered jar and you will only have to scrape off the paraffine when you wish to dispense " white translucent masses." I usually break into lumps about the size of hen's egg and use a four ounce tin ointment box as a container for the paraffine.

### ICHTHYOL.

Ichthyol is always of uniform composition having this formula, $C_{28}\ H_{36}\ S_3\ O_6\ (NH_4)_2$, is a thick brownish liquid containing 15 per cent. easily assimilable sulphur soluble in water. The reason it is thinner in some instances than others is probably due solely to change of temperature; in warm weather ichthyol will be found to be comparatively thin, while in cold weather it will assume a more syrupy consistency. However, this difference in consistency, it is maintained, in nowise impairs the therapeutic action of the drug.—*Merck's M. R. and Phar. Journal.*

PERMANENT SYRUP OF IPECAC.

Mr. Geo J. Geiger writes that the following formula yields an ipecac syrup that will keep a long time.

Fluid extract of ipecac, ℥ ss (15 c.c.)

Loaf sugar, ℥ viii (250 gms.)

Pure water, q. s. ℥ viii (237 c.c.)

Mix the fluid extract with 3½ oz. (104 c.c.) water, shake well and filter. Put the sugar into a percolator, add the filtrate and percolate—adding enough water to make half pint (237 c.c.).—*Merck's Report.*

